# Tunable Release of Ions from Graphene Oxide Laminates for Sustained Antibacterial Activity in a Biomimetic Environment

**DOI:** 10.1002/smll.202304850

**Published:** 2024-04-30

**Authors:** Swathi Suran, Negin Kamyar, Kun Huang, Farzad Foroutan, Premlal Balakrishna Pillai, Xuzhao Liu, John Vaughan, Darren Wilson, Philip J. Day, Rahul R. Nair

**Affiliations:** ^1^ National Graphene Institute & Department of Chemical Engineering University of Manchester Manchester M13 9PL UK; ^2^ Present address: imec The Netherlands High Tech Campus 31 Eindhoven 5656AE Netherlands; ^3^ Department of Materials University of Manchester/Photon Science Institute University of Manchester Manchester M13 9PL UK; ^4^ T. J. Smith and Nephew Limited 101 Hessle Road Hull HU3 2BN UK; ^5^ Manchester Institute of Biotechnology & Division of Evolution Infection & Genomic Sciences University of Manchester Manchester M13 9PL UK

**Keywords:** antibacterial activity, graphene oxide membrane, silver ions, sustained release

## Abstract

Silver has long been recognized for its potent antimicrobial properties, but achieving a slow and longer‐term delivery of silver ions presents significant challenges. Previous efforts to control silver ion dosages have struggled to sustain release for extended periods in biomimetic environments, especially in the presence of complex proteins. This challenge is underscored by the absence of technology for sustaining antimicrobial activity, especially in the context of orthopedic implants where long‐term efficacy, extending beyond 7 days, is essential. In this study, the tunable, slow, and longer‐term release of silver ions from the two‐dimensional (2D) nanocapillaries of graphene oxide (GO) laminates incorporated with silver ions (Ag‐GO) for antimicrobial applications are successfully demonstrated. To closely mimic a physiologically relevant serum‐based environment, a novel in vitro study model using 100% fetal bovine serum (FBS) is introduced as the test medium for microbiology, biocompatibility, and bioactivity studies. To emulate fluid circulation in a physiological environment, the in vitro studies are challenged with serum exchange protocols on different days. The findings show that the Ag‐GO coating can sustainably release silver ions at a minimum dosage of 10 µg cm^−2^ day^−1^, providing an effective and sustained antimicrobial barrier for over ten days.

## Graphical abstract

A slow and longer‐term delivery of silver ions for sustained antimicrobial activity is developed by incorporating silver ions within the 2D nanocapillaries of GO laminates. The controlled release of silver ions from these laminates has provided sustained antimicrobial activity for more than 10 days in a biomimetic environment.

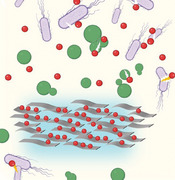

## 1 Introduction

The antibacterial effects of silver (Ag) have been explored for centuries.^[^ ^1^, ^2^, ^3^ ^]^ Metallic silver is more or less inert, but silver elutes tiny fractions of ions in an aqueous solution, making them sparsely bactericidal. A large surface area to volume ratio allows the use of silver nanoparticles (NPs) of various dimensions and shapes as antibacterial nanomaterials.^[^ ^4^, ^5^, ^6^, ^7^ ^]^ However, the rapid and uncontrolled release of Ag^+^ ions from NPs causes unnecessary depletion of resources and accumulation of excess ions in the surrounding cells resulting in severe cytotoxicity.^[^ ^7^, ^8^, ^9^, ^10^ ^]^ Previous research has found that Ag^+^ ions are the most active entity “directly” responsible for the antimicrobial effect,^[^ ^1^, ^11^, ^12^, ^13^, ^14^ ^]^ reducing the required minimal inhibition concentration (MIC) to ≈57% as opposed to Ag NPs.^[^ ^15^, ^16^ ^]^ Ag^+^ ions are found to interfere with the well‐being of the cell by possible synergistic mechanisms, including respiratory chain disruption resulting in reactive oxidative stress (ROS),^[^ ^17^ ^]^ biological dysfunction by binding to the thiol groups of enzymes and proteins in the bacterial cell causing the deoxyribonucleic acid (DNA) to condense and/or physically damaging the cell membrane itself.^[^ ^2^ ^]^ There exist several commercialized pharmaceutical interventions utilizing silver, such as barrier dressings with antibacterial properties. However, they burst release large concentrations of Ag^+^ ions, which can be harmful to the surrounding tissue.^[^ ^9^, ^10^ ^]^ Different silver and non‐silver‐based galvanic couples have been woven into the dressing and implant materials like stainless steel and titanium.^[^ ^18^, ^19^, ^20^, ^21^, ^22^, ^23^ ^]^ Also, the ion exchange process in Zeolite has been explored for the controlled release of Ag^+^ ions.^[^ ^11^, ^12^, ^24^, ^25^, ^26^, ^27^, ^28^ ^]^ However, all of the above technologies either raise regulatory procedural concerns or fail clinical efficacy by not sustaining the MIC.

**Figure 1:**
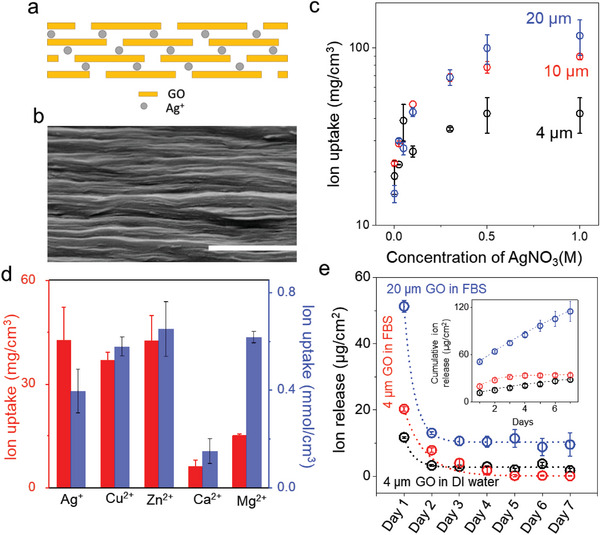
Ion uptake and release in GO laminate. a) Schematic showing Ag^+^ ions incorporated in the interlayer spacing of GO laminate. b) SEM cross‐section of Ag‐GO laminate; scale bar 1 µm. c) Ag^+^‐ion uptake of GO laminate obtained from 4, 10, and 20 µm thick GO laminate as a function of the concentration of AgNO_3_ solution. d) Different cation uptake of 4‐micron‐thick GO laminates was obtained by immersing the laminate in the corresponding 1 m salt solution for 24 h. e) Ag^+^ ion release rate into DI water and FBS media from 4 and 20 µm thick GO laminates. 20 µm thick GO laminate delivers a sustained release of >10 µg cm^−2^ day^−1^ on average until day 7. Inset; cumulative ion release as a function of the number of days. Error bars denote the standard deviation from three samples.

Recently, graphene and related two‐dimensional (2D) materials have been explored as supports for hosting various nanomaterials, such as metals, metal oxides, and polymers, exhibiting high antibacterial efficiency.^[^ ^29^ ^]^ Most of these efforts have focused on preparing composites of NPs and graphene‐related materials (e.g., GO and Ag NPs) to demonstrate their antimicrobial properties. The AgNPs in these studies are prepared by reducing Ag^+^ ions to AgNPs, but during these processes, GO also gets reduced to reduced GO (RGO). Various characterizations, including X‐ray diffraction (XRD) and Transmission Electron Microscopy, clearly show the presence of AgNPs in such systems. However, such materials do not allow for the controlled release of Ag^+^ ions over an extended period because these composite materials contain macroporous structures (primarily due to the reduction process) that permit the diffusion of other molecules (e.g., proteins) into the composites, leading to a deterioration of their antimicrobial properties. To address this issue and prevent the potential overdosage of AgNPs, we have adopted a novel approach. This approach involves incorporating Ag^+^ ions into the well‐defined 2D capillaries formed by closely spaced GO sheets within GO laminates (**Figure** **1**a,b) and releasing them in a controlled manner over an extended period.

GO laminate recently attracted intense interest in tunable membranes for ion separation and filtration technology.^[^ ^30^, ^31^, ^32^, ^33^, ^34^ ^]^ Due to the negative surface charge of GO sheets, membranes made of GO can adsorb a significant amount of cations into their interlayer space (Figure 1a).^[^ ^34^, ^35^, ^36^ ^]^ In aqueous environments, GO develops a negative charge due to the deprotonation of carboxylic functional groups. This charge imbalance encourages the adsorption of ions from the surrounding liquid, thus maintaining the overall electroneutrality and structure of the laminates. It is worth noting that this scenario differs for Ag nanoparticle‐GO composites, as the incorporation of Ag NPs alters the structure of the GO membranes and significantly reduces the surface area. The ion uptake primarily depends on the surface charge density of GO, the diameter of hydrated ions, and their charge. This property of GO laminate has been explored for radioactive ion capturing and water filtration applications.^[^ ^34^, ^35^ ^]^ These laminates are also capable of exclusively transporting water and ions between the layers while effectively blocking larger molecules, including proteins. This unique property allows for the stable storage of ions within the GO capillaries for extended periods, enabling sustained release driven by concentration gradients. Here, we demonstrate that the adsorbed ions can be controllably released not only into pure water but also into a physiologically relevant serum environment (100% serum) by studying Ag^+^ ion release dynamics. Furthermore, we showed sustained antimicrobial activities of Ag‐GO laminate‐coated polyester (PE) sheets (to mimic wound dressing material) and titanium (Ti) rods (to mimic an orthopedic implant) by using *Staphylococcus aureus* as a bacterial model. We define and demonstrate a new serum challenge protocol that closely mimics the pathophysiological scenario to test the antibacterial activity. To emulate physiological fluid circulation/exchange, we challenged our samples by applying fresh bacterial‐serum cultures at different time points of the study. Despite multiple serum challenges, we observed a 4‐log reduction in the bacterial suspension counts with sub‐100 surface adherent bacteria for more than 10 days of testing. It is noteworthy that the methodology we employed, utilizing 100% fetal bovine serum (FBS) with frequent serum changes to mimic body fluid flow, has not been reported in any other publications. Additionally, we have also performed cytotoxicity and bio‐activity studies, showing that the developed coating is biocompatible.

## 2 Results and Discussion

GO laminates prepared via vacuum filtration were immersed in AgNO_3_ solution for 24 h to allow the incorporation of Ag^+^ ions into the GO laminates. The resulting Ag‐GO laminates were then thoroughly rinsed in water to remove all the surface‐adsorbed silver. Figure 1a shows the schematic structure of the Ag‐GO laminate. The very fact that no AgNPs  were visualized in the SEM cross‐section images indicates that only silver ions are captured in the interlayers of GO (Figure 1b). This is further confirmed by scanning transmission electron microscopy and XRD (see Section S1, Figure S1, and S2, Supporting Information). XRD also confirms that Ag^+^ adsorption into the GO laminate does not alter the laminar structure of the membrane in both air and water (Figure S2, Supporting Information).

Figure 1c shows the Ag^+^ ion uptake of the GO laminate as a function of AgNO_3_ concentration, obtained using three different thicknesses of the laminate. All the GO laminates approach Ag^+^ ion absorption saturation when treated with ≈0.3 m AgNO_3_ solution, and the maximum ion uptake capacity is found to be ≈100 mg cm^−3^. For the 4‐micron‐thick laminates, the ion uptake capacity is found to be ≈2.5 times smaller. This could be due to the loss of some of the adsorbed silver from the capillaries during the rinsing of the membrane since thinner samples tend to lose relatively more silver than the thicker samples. This experiment further suggests that the total amount of silver adsorbed into the laminate can be tuned by controlling the thickness of the laminates.

Aside from Ag^+^ ions, we also investigated the absorption properties of other ions of biological interest, which could potentially find applications in areas such as antimicrobial coatings, bone regeneration, and osseointegration, among others. Figure 1d illustrates the maximum ion sorption capacity of GO laminates for Ag^+^, Cu^2+^, Zn^2+^, Ca^2+^, and Mg^2+^ ions (see Methods). To demonstrate the potential application of ion‐adsorbed GO laminates in biomedical contexts as an ion reservoir, we initially examined the ion‐release kinetics of Ag‐GO laminates. Generally, the kinetics depend on the interaction between silver ions and GO as well as the concentration gradient. However, in this study, we utilized a serum medium for ion release, and the interaction between ions and the serum will also impact the release kinetics.

The release of Ag^+^ ions from GO laminates (per unit surface area) was investigated in deionized (DI) water and a whole serum medium (FBS) at 37 °C over seven days. Figure 1e presents the Ag^+^ ion release profile over time, analyzed using inductively coupled plasma optical emission spectroscopy (ICP‐OES) (see Experimental Section). A rapid burst release of ions was observed at the end of the first day, after which the ions exhibited a steady, constant release in the subsequent days. Compared to DI water, the burst release of ions in a serum environment was found to be larger (≈2 times greater). The steady‐state ion release rate in FBS from Ag‐GO laminate was found to be adjustable with the thickness of the laminate. For instance, a 4 µm thick laminate provided an average of ≈0.2 µg cm^−2^ day^−1^ of Ag^+^ ion release, whereas a 20 µm laminate provided an average of ≈10 µg cm^−2^ day^−1^ until day 7. This suggests that the sustained release of Ag^+^ ions from Ag‐GO laminate could be controlled by varying the thickness of the laminate.

**Figure 2:**
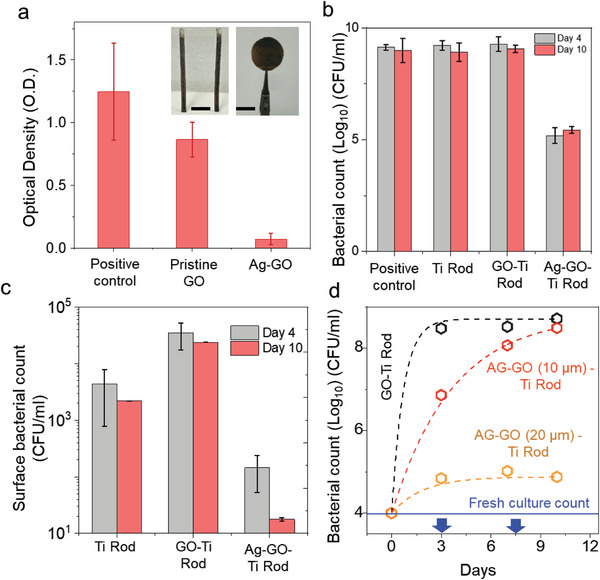
Antibacterial properties of Ag‐GO laminates. a) The optical density of bacterial cultures was measured after 48 h of culture in the presence of pristine GO‐coated PE and Ag‐GO‐coated PE. Positive control without any sample (culture alone) is also shown. Inset: photographs of Ag‐GO coated Ti rod (left), and Ag‐GO coated PE (right). Scale bars: 1 cm. b) Bacterial suspension counts with 20 µm thick Ag‐GO coated Ti rods with respect to control samples measured on day 4 and day 10. c) Surface bacterial count from 20 µm Ag‐GO coated Ti rods with respect to control samples measured on day 4 and day 10. d) Bacterial growth kinetics were measured up to 10 days in the presence of a GOTi rod, Ag‐GO (10 µm) Ti rod, and Ag‐GO (20 µm) Ti rod. Culture media were replaced on day 3 and day 7, as indicated by blue arrows. The solid blue line shows the fresh bacterial culture count. Dashed lines are a guide to the eye. The larger bacterial count from 10 µm AG‐GO coated Ti rod is due to the depletion of Ag^+^ ions with time, whereas in 20 µm, the Ag loading is high, a sustained release and hence a lower bacterial count is observed. Error bars denote the standard deviation from four samples.

To explore the antimicrobial effects of released Ag^+^ ions, we studied the antimicrobial activities of Ag‐GO laminate‐coated PE (to mimic wound dressing material) and Ti rods (to mimic orthopedic implant) (**Figure** **2**a inset and methods). We primarily used *S.aureus*, strain Newman, in this study since it is one of the most common yet prevalent bacteria causing infections in orthopedic and topical wounds alike.^[^ ^37^, ^38^ ^]^ 100% FBS serum was chosen in this study to mimic the physiological conditions more closely than common microbiological media or diluents. The presence of thiol‐containing amino acids (in the protein constituents) in the media containing FBS causes silver to form silver thiolate complexes in contrast to the typical culture media like Luria–Bertani (LB) broth which only had non‐sulfur containing amino acids.^[^ ^39^, ^40^ ^]^ The protein pool in the human serum and the prevalence of cation–protein interactions results in decreased bactericidal activity, interfering with the susceptibility tests. It is noteworthy that due to the Ag^+^ ion‐sulfhydryl complexation, a sustained release of antimicrobial agents for a prolonged time (e.g., more than 7 days) is challenging and has not been achieved so far.

A quick qualitative study of the antibacterial performance of our test samples was carried out by disk‐diffusion or the Kirby Bauer test with *S. aureus*, strain Newman on LB agar solid media to observe the zone of inhibition (ZoI) (See Section S2, Supporting Information). These studies suggest that Ag‐GO laminates are antibacterial, whereas pristine and Cu/Zn ion adsorbed GO were not antibacterial. To further confirm the antibacterial properties of Ag‐GO, we performed optical density (O.D) measurements following culture in liquid media to study the turbidity, and therefore growth, of the bacterial culture. Ag‐GO (4 µm thick) coated PE was tested for its antibacterial performance with respect to the primary and secondary controls, which were “no sample, only bacteria in growth media” and “GO‐PE without Ag^+^ absorption,” respectively. The optical density (OD) or absorption of the bacterial culture measured following 48‐h incubation, shown in Figure 2a, suggested that Ag‐GO‐coated PE has bactericidal properties compared to GO‐coated PE. An OD value of 0.1–0.2 for a bacterial culture with Ag‐GO‐coated PE corresponds to bacterial counts of ≈10^4^–10^5^ Colony Forming Units (CFU) mL^−1^. However, the OD value for GO‐coated PE was measured at ≈1.25 and 0.8, respectively, which further suggested the bactericidal properties of Ag‐GO‐coated PE compared to GO‐coated PE. To understand the effect of substrate (PE) on the properties of Ag‐GO, we compared the antibacterial performance of free‐standing Ag‐GO laminate and Ag‐GO coated PE and found that both had similar performance (Section S3, Supporting Information).

To further probe the sustained antimicrobial effects for an extended period and potential applications on orthopedic implants, we made Ag‐GO coatings on Ti rods, as shown in Figure 2a inset. These coatings are prepared by a simple layer‐by‐layer deposition process (methods). For an efficient antibacterial performance for an extended period of time, Ag^+^ loading on these coatings was increased by increasing the thickness of the GO coating to 20 µm. Figure 2b shows the bacterial suspension count measured by cell plate counting from samples with 20 µm thick Ag‐GO coated Ti rods, which will be referred to as 20‐AgGOTi rods in the text. A starting bacterial count of 10^4^ CFU mL^−1^ was used to emulate an orthopedic surgical site scenario and was maintained for all experiments, with controls being only bacterial culture without sample, bare Ti rod, and a GO‐coated Ti rod. As seen in Figure 2b, the bacterial counts in suspension were measured on days four and ten, following bacterial culture renewal on days three and seven, respectively. To our surprise, the 20‐AgGOTi rods continued to display activity following both culture changes resulting in bacterial counts three orders of magnitude lower (10^5^ CFU mL^−1^) in comparison to the controls with bacterial counts of 10^9^ CFU mL^−1^ as counted from agar plates. Adhered bacteria to the surface of the samples were enumerated by performing a phosphate‐buffered saline (PBS) rinse protocol. The samples were carefully removed from the suspension and rinsed in PBS for 1 min at 50 rpm. This step was repeated three times, followed by manual agitation of the samples to ensure complete removal of loosely bound bacteria from sample surfaces prior to enumeration of surface adhered bacterial count. The third PBS rinse elute was plated and counted from agar plates‐undiluted volumes (neat) from the test samples and respective diluted volumes for the control samples. Results showed that the test sample 20‐AgGOTi rod had less than 100 bacteria per sample as compared to 1000–10 000 bacteria per control sample. This suggested that the Ti rod coated with Ag‐GO strongly inhibited bacterial growth on the surfaces and reduced the bacteria suspension count in the surrounding solution.

To demonstrate the importance of a tunable amount of Ag^+^ loading on the observed long‐time antibacterial property, we studied the bacterial growth kinetics for up to ten days using a bare Ti rod, 10 µm thick Ag‐GO coated Ti rod (10‐AgGOTi), and 20 µm thick Ag‐GO coated Ti rods (Figure 2c). The culture media were changed on days three and seven to mimic the fluid/serum circulation in physiological conditions, and this caused a significant loss of ions released into the media. Growth of bacteria to 10^9^ CFU mL^−1^ was observed for a bare Ti rod (from a starting count of 10^4^ CFU mL^−1^), whereas the test samples, 10‐AgGOTi and 20‐AgGOTi, allowed a bacteriostatic growth of up to 10^7^ and 10^5 ^CFU mL^−1^, respectively, on day 3. Interestingly, it was observed that the sample 20‐AgGOTi rod continues to inhibit bacterial growth, maintaining < 10^5^ CFU mL^−1^ until day 10, unlike the control Ti rod or the 10‐AgGOTi, which reaches a maximum bacterial count of 10^9^ CFU mL^−1^. Hence, the 20‐AgGOTi rod with a higher Ag^+^ ion loading has a 10‐day antibacterial efficacy owing to the sustained release rate of Ag^+^ ions despite the stringent serum challenge.

Further, we studied the viability or culturability of bacteria that had been exposed to the Ag^+^ ions eluted from the test samples (Section S4, Supporting Information). Our study found no significant viability or culturability for the bacteria exposed to Ag^+^ ions. Such a state of the population is known as the Alive but Non‐culturable (ABNC) population (Section S4, Supporting Information). This indicates that the exposure of Ag^+^ ions from the GO laminates may not induce resistance to silver ions in bacteria. Despite previous studies showing no evidence of resistance to Ag^+^ ions, even with prolonged ion exposure for 42 days,^[^ ^41^ ^]^ additional dedicated studies are required to explore any potential bacterial resistance to Ag^+^ ions released from the GO laminates.

**Figure 3:**
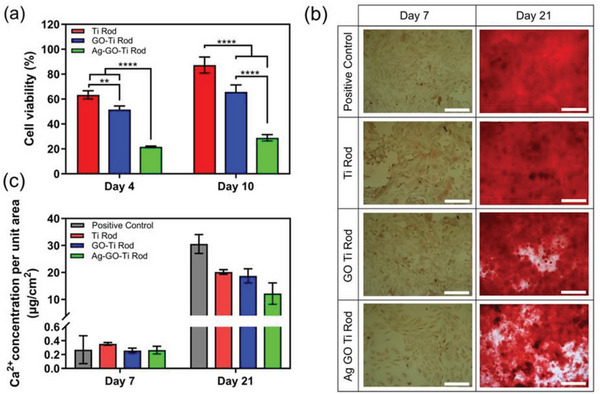
Cytotoxicity and calcification studies. a) Cell viability percentage of Saos‐2 cell for AgGO (20 µm) coated Ti rod, GO (20 µm) coated Ti rod, and Ti rod. Cell viability of Saos‐2 cells was studied in 100% FBS to emulate the antibacterial study conditions. The comparisons between experimental groups were assessed using ANOVA with Tukey's test using GraphPad Prism software (*p*‐values: *p < 0.05, **p < 0.01, ***p < 0.001, and ****p < 0.0001). b) In vitro analysis of calcium deposition of Saos‐2 cells cultured in osteogenic media was measured using Alizarin Red S Staining Assay. (Scale bars = 250 µm). c) Quantitative analysis of calcium deposition of Saos‐2 cells using ICP‐OES. Increased calcium deposition was observed between day 14 and day 21 for all the samples.

To study the biocompatibility of the developed Ag‐GO coating, we performed cytotoxicity studies by using Saos‐2 cells. We used positive control, Ti rod, and 20‐AgGOTi rod samples for these studies. The cellular proliferation of Saos‐2 cells with indirect contact with Ti rods was assessed using the PicoGreen assay on days 4 and 10 in FBS. **Figure** **3**a shows the cell viability % relative to the number of cells in the positive control without the presence of samples at each specific time point. We show that on day 4, the cell viability% (see Experimental Section) for the 20‐AgGOTi is ≈22%, increasing to 29% on day 10, confirming osteoblast cell recovery. The smaller cell viability for 20‐AgGOTi on day 4 could be attributed to a high release amount of Ag^+^ ions during the first 2 days, as shown in Figure 1e. On the other hand, even on day 10, bacterial viability is <0.02%, demonstrating the longer‐term antibacterial efficacy of the Ag‐GO coating with relatively lower cytotoxicity.

Since the developed coating has potential applications in orthopedic implants, we have also studied the effect of Ag^+^ ions on calcification. The extracellular matrix calcification of Saos‐2 cells with in‐direct contact with 20‐AgGOTi rods, GO Ti rod, and Ti rods was analyzed using Alizarin Red staining (ARS). ARS staining is a qualitative assay for assessing the deposited calcium metal ions as a marker for bone mineralization.^[^ ^42^ ^]^ Figure 3b shows ARS images of Saos‐2 cell culture on days 7 and 21. On day 7, all samples showed a successful cellular proliferation of Saos‐2 cells; however, no calcification was observed. The Saos‐2 cells showed a significant increase in calcium deposition following 21 days of culturing for all groups of samples (Figure 3b). For quantitative analysis of the deposited calcium, the stained samples were digested in a 70% nitric acid solution, and the calcium concentration per unit area was measured using ICP‐OES, as shown in Figure 3c. In agreement with ARS staining, very low calcium deposition (0.24–0.35 µg cm^−2^) was observed on day 7 for all the samples. On day 21, a lower calcium deposition for 20‐AgGOTi (≈12 µg cm^−2^) was obtained compared to control GOTi rod and Ti rod samples (≈20 µg cm^−2^). By comparing the toxicity and calcification studies (Figure 3a,b), an increase in calcium deposition for 20‐AgGOTi from day 7 to day 21 further confirmed the recovery of cell proliferation after day 10. Although 20‐AgGoTi showed lower cell proliferation and calcification than in control samples, the formation of calcium suggests that physiological issues caused by the controlled release of Ag^+^ ions are transient and unlikely to cause significant issues across the lifetime of the implants.

## 3 Conclusion

In conclusion, we report a sustained and controllable release of antibacterial Ag^+^ ions using GO laminates with nanometer interlayer capillaries. We demonstrate that the loading of Ag^+^ ions can be adjusted as needed by varying the thickness of the GO, and the loaded Ag^+^ ions can be released in a controlled manner into physiologically relevant media. The slow release of Ag^+^ ions into the media, driven by the concentration gradient, can be attributed to the ultralong tortuous GO capillary (≈20 mm long for a 20 µm thick laminate). The tunability in Ag^+^ ions uptake and release allows us to use this laminate on PE films and Ti rods as an antibacterial coating. Despite multiple serum challenges, our coatings retain antibacterial efficacy for up to 10 days with a minimum sustained Ag^+^ ion release of 10 µg cm^−2^ day^−1^. Moreover, these coatings exhibit minimal cytotoxicity and support mineralization (calcification) on the surface of the implants, which can initiate osseointegration. Our work introduces a new strategy for developing tunable nanocapillary‐based coatings for the controlled and sustained release of bioactive ions and molecules.

## 4 Experimental Section

### Preparation of Free‐Standing GO Laminates

Free‐standing GO laminates were prepared using ultrasonic exfoliation, centrifugal separation, and vacuum filtration techniques, as reported previously.^[^ ^30^, ^31^ ^]^ GO pellets, commercially procured from William Blythe, were suspended in deionized (DI) water at a concentration of 5 mg mL^−1^ and subjected to ultrasonication for 8 h. The resulting dispersion was centrifuged at 8000 rpm for 1 h to remove very small‐sized GO flakes. The pellet was then resuspended in DI water and centrifuged at 8000 rpm for 20 min to collect the supernatant consisting of GO. This two‐step process ensured a homogeneous mix of equally sized flakes with a GO suspension concentration of 1.5 ± 0.2 mg mL^−1^. A 20‐fold dilution of this GO suspension was used to prepare free‐standing GO laminates by vacuum filtration technique. Anodic alumina (Whatman filters, 0.2 µm pore size, 25 mm diameter) filters were used as a support membrane for GO vacuum filtration. The resulting GO laminates were air‐dried at 60 °C for 12 h. The thickness of the free‐standing GO laminates was varied by filtering different volumes of GO suspension.

### GO Coating on Ti rod and PE Disc

To demonstrate the technology in implantable devices, titanium rods of purity >99.8%, temper annealed, 5 cm in length, and 0.2 cm in diameter, were procured from Advent Research Materials Ltd., United Kingdom, and used as substrates to coat GO. The Ti rods were first rinsed with acetone, ethanol, and ultrapure water successively and then air‐dried at 60 °C for 2 h. To improve surface adhesion, the Ti rods were treated with corona discharge for 2 min in the open air at 20 °C and atmospheric pressure, with a power output of 180 W. For the preparation of the GO coating solution, the 1.5 mg mL^−1^ GO solution was centrifuged at 12 000 rpm for 2 h, followed by removing the supernatant. The sediment was re‐dispersed in deionized water to obtain a GO concentration of 7.5 mg mL^−1^ for coating the Ti rods. A multistep dip‐coating procedure (DIP COATER QPI‐168, Qualtech Products Industry) was customized. After optimization of the immersion and withdrawal rates during dip coating, the Ti rods were immersed at a speed of 500 mm min^−1^ for 5 s and withdrawn at a speed of 500 mm min^−1^. Through this step, a very thin layer of GO was allowed to adhere sequentially to the surface of the Ti rods. The entire coating process was carried out at 20 °C. After each round of coating, the coated Ti rods were dried in an air oven for 30 min at 60 °C before allowing further coating. To ensure the uniformity of the coated layer, after each phase of coating and drying, the Ti rods were turned upside down for the subsequent coating step. Finally, the GO‐coated Ti rods were dried in an air‐circulating oven at 60 °C for 12 h to remove the residual water and ensure the formation of a GO coating layer. Other biocompatible substrates like polyester (Drain disc, Thickness: 100 µm, GE Healthcare) were also used for GO coating. For this purpose, the polyester disks were immersed in GO dispersion (7.5 mg mL^−1^) and dried in the incubator for 30 min to allow uniform coating (the coating was repeated three times in total).

### Ion Loading into GO Laminate and Coating

To incorporate different metal ions into the nanocapillaries of the GO laminates and coatings, nitrate salts of Ag^+^, Cu^2+^, Zn^2+^, Ca^2+^, Mg^2+^, and Sr^2+^ were used. All salt solutions were prepared using ultrapure water. To allow diffusion of ions into the GO laminate, the free‐standing GO laminates were kept afloat on 10 mL of 1 m aqueous salt solutions for 24 h at a temperature of 25 °C, with continuous shaking at 50 rpm. After the metal ion uptake, the treated GO laminates were removed from the salt bath and placed in ultrapure water to wash the surfaces and minimize surface contributions while maintaining a shaking speed of 100 rpm for 20 s, repeated twice. The ion‐incorporated GO laminates were then air‐dried in an oven at 60 °C for 12 h. To measure the ion uptake, the laminates were digested by ashing in a laboratory air‐circulating chamber furnace (Carbolite Gero) at 650 °C for 2 h. The ashed residues were then digested in 70% HNO_3_ at 300 rpm for 3 h before measuring the ion concentration in ICP‐OES.

To incorporate Ag^+^ ions into the GO coatings of the Ti rod, and PE film, the coated materials were treated with a 1 m aqueous solution of AgNO_3_ for 24 h at 25 °C with continuous shaking at 50 rpm.

### Ion Release from GO Laminate and Coating

For ion release studies, free‐standing GO laminates were immersed in 5 mL of ultrapure water or FBS at 37 °C with constant agitation of 50 rpm. The release study was carried out at different time points with a change/refill of media at these respective time points‐ from day 1 to day 7. The solutions withdrawn at each time point were further digested in 2 mL 70% HNO_3_ followed by dilution in 8 mL deionized water and agitated at 300 rpm for 3 h to quantify metal ions released in the respective media using ICP‐OES.

### Quantification of Ion Concentrations in ICP‐OES

An inductively coupled plasma optical emission spectrometer (ICP‐OES, PlasmaQuant PQ 9000 Elite, Analytik Jena AG, Germany) was used to measure the ion concentrations. Solutions were introduced into the plasma (at 1200 W) chamber using an ASPQ3300 autosampler under a continuous flow of argon gas. Samples were measured in quadruplicate using an axially positioned quartz torch, a double monochromator, and a high‐resolution charge‐coupled device (CCD) detector. The quantitative analysis for elemental content was made against the five‐point calibration curve. Calibration curves were established by plotting the mass‐uncorrected counts as a function of the concentration of element (in mg L^−1^) and performing linear least squares regression on the plot. The signals for the elements were monitored with the most intense emission wavelength at 328 and 338 nm. The ion concentrations quantified and reported here were from the emission line at 338 nm.

### Antibacterial Studies

Unless mentioned otherwise, all the chemicals and consumables were purchased from Sigma (Sigma Aldrich, UK; now Merck, UK). *Staphylococcus aureus* (*S.aureus* strain‐Newman) was provided by Prof R. Goodacre, University of Liverpool.^[^ ^43^ ^]^ The bacterial strains frozen at 80 °C were thawed into nutrient agar media which consisted of a mixture of peptone 5 g L^−1^, yeast extract 3 g L^−1^, agar 15 g L^−1^, and distilled water. The streaking on solid nutrient agar plates was repeated twice before transferring to the liquid culture. Then, a single colony was transferred to a 50 mL conical flask containing 15 mL of FBS and was grown at 37 °C with continuous shaking at 200 rpm in a standard shaker incubator (New Brunswick Scientific). All cultures used in the experiments mentioned in this paper were sampled from cultures grown for 16–18 h, which were 2–4 h into their stationary phase. For bactericidal testing, the as‐grown bacterial cultures, which were 2–4 h into the stationary phase, were diluted 1000‐fold. Negative control was always uninoculated FBS media and positive control was *S.aureus* cultured in FBS without any samples. All the GO samples were subjected to 3 mL of bacterial culture of a starting count of 10^4^ CFU mL^−1^ in 15 mL Falcon tubes for the Ti rod samples and 50 mL Falcon tubes for GO free‐standing laminates or GO‐coated PE films, owing to their dimensions staying fully immersed in the cultures during the entire length of study. All bacterial counts were measured by serial dilution of suspension solution by the traditional plate counting methodology on LB agar plates.

To enumerate bacterial counts by iQue 3.0 IntelliCyt flow cytometer (Sartorius, UK), suspension solution was appropriately diluted in PBS. Flow cytometry (FC) allowed bacterial enumeration on the same day to map a picture of the full growth kinetics as the eluted ions were lost due to subsequent culture changes. Hence, FC was occasionally used to tally some of the bacterial counts with the counts from the plates. A membrane potential sensitive slow dye, disc3(5), conjugated only to live bacterial cell membranes. The starting count of the bacteria was maintained at ≈10^4^ CFU mL^−1^. The volume sampled by the flow cytometer was fixed using a Sip time‐2 s (actual sample uptake) and pump speed‐29 rpm (which corresponds to 1.5 µL s^−1^ sample uptake) for a U‐bottom 96‐well plate model. The data reported were averaged from 4 Nos. of each type of sample with four replicates each. The buffer solution used for the flow cytometer runs was PBS with 1/1000× of the proprietary QSol buffer and 0.1% of BSA. This mixture made sure of no cell aggregation during cell counting. The fluorescent dye used to tag bacterial cells for cytometry was DiSC3(5)[3,3‐dipropylthiadicarbocyanine iodide], procured from AAT Bioquest. All fluorescence measurements with excitation at 640 nm and detection in RL1 channels at 675 nm. Well identification, gating, and analysis were performed in the software ForeCyt provided by Sartorius. Bulk OD measurements if and when performed, were in Corning F‐bottom 96‐well plates (Corning, UK) with three replicates of each sample.

### Protocol for Bacterial Surface Counts

Unlike bacterial suspension count, the bacteria on the surface of the rods were to be carefully isolated from the loosely bound bacteria. A protocol with triple rinsing in PBS was developed. The rods were carefully removed from the PBS solution and rinsed in DI water. The samples were then agitated for 1 min at 50 rpm sequentially three times in PBS with intermittent rinse steps in the water. The bacterial counts (measured by cell plate counting) obtained from the third PBS rinse were used to report the surface‐bound bacterial counts owing to the consistency of results from all sample sets.

### Materials for Cell Culture Studies

Dulbecco's modified Eagle's medium (DMEM, 4500 mg L^−1^ glucose, D5796), minimum essential medium (α‐MEM, M4526), fetal bovine serum (FBS, F9665), antibiotics (10 000 units mL^−1^ penicillin, 10 000 µg mL^−1^ streptomycin and 25 µg mL^−1^ amphotericin), β‐glycerophosphate disodium salt hydrate (BGP, G9891), l‐ascorbic acid 2‐phosphate sesquimagnesium salt hydrate (Asc‐2‐phos, A8960), dexamethasone (D8893), hydrochloric acid (320331), formalin solution 10% (HT501128), and Triton‐X100 (X100‐5 mL) were purchased from Sigma–Aldrich, UK. Quant‐iT PicoGreen dsDNA Assay Kit (P7589), Gibco GlutaMAX Supplement (35050038), and ammonium hydroxide (10470481) were purchased from ThermoFisher Scientific, UK. 20X TE Buffer (A2651) was purchased from Promega, USA. Alizarin Red S (HD1055) was purchased from TCS Biosciences. 12 well thincert (0.1 µm pore diameter, 665610) was purchased from Greiner Bio‐One Limited, UK. The coverslips (Round, diameter 20 mm, 6311581) were purchased from VWR International, UK.

### Cell Cytotoxicity Study

For the indirect cell viability study, the Saos‐2 cells at the density of 2 × 10^6^ were cultured in 100% FBS in a T75 flask. Once the cells reached 80% confluency, 80 µL of cell suspension with a density of 30 000 Soas‐2 cells was seeded on a coverslip placed in 12 well plates. Triplicated of each UV sterilized Ti, GOTi, and AgGOTi rods were placed in cell thincert and transferred to the 12 well plate containing adhered cells to the coverslip. 3 mL of PBS was added to each well plate, and the cells were incubated for 21 days. PicoGreen fluorescence assay was used to analyze the cell viability on days 4 and 10. At each day point, the cell thincerts containing the rods were removed from each well plate, and the cells were washed with PBS. The cells were then resuspended in 1 mL of 1× lysis buffer (0.2% Triton X‐100 in 1× TE buffer solution) and freeze–thawed thrice. A 100 µL (three replicates) of each cell lysed solution was added to 96 well plates, followed by the addition of 100 µL of 200‐fold Picogreen solution. The samples were incubated for 5 min, and the fluorescence intensity was recorded at 480/520 nm wavelengths. The cell viability % for AgGO (20 µm) coated Ti rod, GO (20 µm) coated Ti rod, and Ti rod on days 4 and 10 were calculated based on the control condition (cells seeded without samples) as given by the Equation 1 below:

(1) $$Cell\;viability\;\%=\left(1-\frac{\;Ax-Bx}{Ax}\;\right)\times 100$$

where, *A*x is the average number of live cells for the control condition on day x and *B*x is the average number of live cells for Ti rod samples on day x. The statistical significance was carried out using two‐way ANOVA with Tukey's post hoc analysis test.

### Samples Preparation and Saos‐2 Cell Seeding for Alizarin Red S Staining Assay

For the preparation of osteogenic medium, 500 mL α‐MEM was supplemented with 10% v/v FBS, 1% v/v antibiotics, 5× 10–7 mm dexamethasone, 10 mm BGP, and 100 µm Asc‐2‐phos and 1× GlutaMAX. For the cell indirect contact study, the sterilized coverslips were placed in 12 well plates and immersed in a standard medium for 24 h to wet and precondition the CV prior to cell seeding. 80 µL of the cell suspension at a density of ≈30 000 Saos‐2 cells were seeded on CVs in triplicates and incubated for 1 h to allow cell adhesion before adding 3 mL osteogenic media. Then each UV sterilized Ti, GOTi, and AgGOTi rods were placed in cell thincert and transferred to the well plate containing adhered cells to the coverslip. The cells seeded on coverslips without any Ti rod samples were used as a positive control.

Alizarin Red S staining assay was carried out for qualitative analysis of calcium deposition of Saos‐2 cells. For this purpose, 2 g of Alizarin red S powder was dissolved in 100 mL DI water, and the pH was adjusted to ≈4.1 using hydrochloric acid or ammonium hydroxide (Fisher Scientific, 10470481). The obtained solution was filtered (0.22 µm) and stored at room temperature in the dark prior to use. On days 10 and 21, the cells were removed from the incubator, and the thincerts containing Ti, GOTi, and AgGOTi rods were removed from the well plate. Then the cells were washed with PBS and were fixed using 10% formalin for 30 min. Then the formalin solution was aspirated carefully, and the cells were washed with di‐water. 1 mL of Alizarin red S staining solution was added to each well plate, and the cells were incubated at room temperature in the dark for 15 min. The Alizarin red S staining was aspirated carefully, and the cells were washed with di‐water four times. The cellular calcium deposition was immediately analyzed using the light microscope (DM4 B Leica microsystems, UK).

### Measuring the Calcium Concentration in ICP‐OES

The calcium concentration of Alizarin Red S‐stained samples was further analyzed using ICP‐OES. For this purpose, 2 mL of 70% nitric acid solution was added to each well plate and left for 5 min to allow digestion. Then the solution was transferred to a 15 mL centrifuge tube and diluted with 8 mL of deionized water, and the calcium concentration was measured using ICP‐OES.

## Conflict of Interest

The authors declare no conflict of interest.

## Supporting information

Supporting Information

## Data Availability

The data that support the findings of this study are available from the corresponding author upon reasonable request.
