# Supplementary material for: Tunable Release of Ions from Graphene Oxide Laminates for Sustained Antibacterial Activity in a Biomimetic Environment
Source: Small. 2024 Apr 30;21(28):2304850. doi: 10.1002/smll.202304850 (PMC12272042; doi:10.1002/smll.202304850)
Supplement: Supplementary file 1 — Supporting Information [file SMLL-21-2304850-s001.pdf]

NANO · MICRO
small

Supporting Information

for *Small*, DOI 10.1002/smll.202304850

Tunable Release of Ions from Graphene Oxide Laminates for Sustained Antibacterial Activity in a Biomimetic Environment

Swathi Suran, Negin Kamyar, Kun Huang, Farzad Foroutan, Premlal Balakrishna Pillai, Xuzhao Liu, John Vaughan, Darren Wilson, Philip J. Day and Rahul R. Nair**

Supporting Information

Tunable release of ions from graphene oxide laminates for sustained antibacterial activity in a biomimetic environment

Swathi Suran, Negin Kamyar, Kun Huang, Farzad Foroutan, Premlal Balakrishna Pillai, Xuzhao Liu, John Vaughan, Darren Wilson, Philip Day, Rahul R. Nair**

1. Transmission Electron Microscopy (TEM) and X-Ray diffraction characterisation of Ag-GO laminates.

Transmission Electron Microscopy (TEM) and Scanning Transmission Electron Microscopy (STEM) imaging were conducted using a FEI Talos F200A FEG-TEM operating at 200 kV and equipped with four Super-X Energy Dispersive X-ray (EDX) detectors. TEM images were acquired using low-dose settings to minimize beam-induced reduction. High-angle annular dark-field (HAADF)-STEM images and STEM-EDX results were acquired using a probe current of 260 pA, and the probe size was estimated to be approximately 1.2 nm. The analysis of the TEM and STEM data was performed using Velox software.

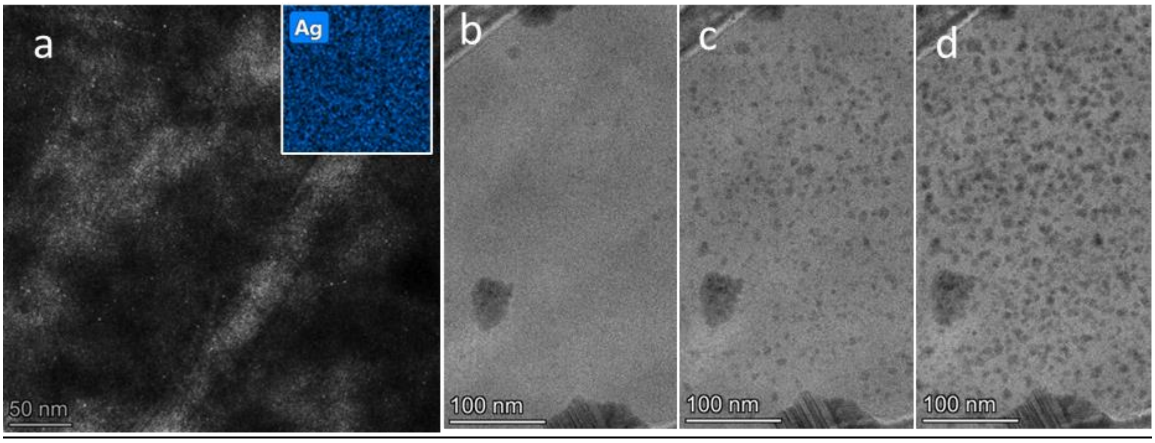

Figure S1: HAADF STEM characterization of Ag-GO laminates. a) HAADF STEM image of Ag-GO membrane deposited on a TEM grid. Inset shows EDX mapping of silver, showing the uniform distribution of silver on the entire surface of the membrane. b-d) TEM images showing the formation of the Ag nanoparticles in Ag-GO membrane upon exposure to the electron beam for 2s (b), 4s (c), and 6s (d).

TEM/STEM analysis was carried out on a 4 µm thick Ag GO membrane after crushing it into a powder and depositing it onto a TEM grid. Figure S1 shows the STEM image of an Ag-GO laminate. The clear, featureless images confirm that the adsorbed Ag is not in nanoparticle form; rather, it remains in ionic form. To confirm the presence of Ag on the membrane, we performed EDX mapping, which shows a uniform distribution of Ag (0.61 at%) throughout the membrane (see inset of Figure S1a). However, after exposing the samples to the electron beam for 2 seconds or longer, we observed the in-situ formation of Ag particles/platelets on the membrane (Figure S1c-d). This could be attributed to the reduction of adsorbed Ag^+ ions into Ag by the electron beam. These experiments confirm that the adsorbed Ag on the Ag-GO membranes is in the ionic state and not in nanoparticle form.

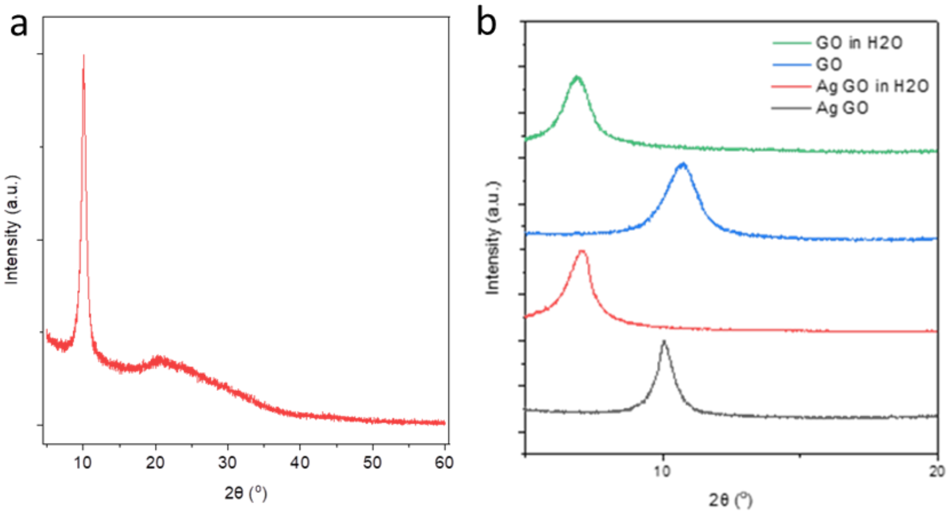

Figure S2: XRD characterisation of Ag-GO laminate. a) XRD from Ag-GO laminate showing the absence of any additional peak due to metallic silver. b) XRD pattern from GO, Ag-GO, and both membranes exposed to liquid water.

To further confirm this, we examined the structure of the laminate using X-ray diffraction (XRD). XRD experiments were conducted with a Rigaku SmartLab XRD system using Cu Kα radiation (λ = 0.154 nm). Figure S2a displays the XRD results for the Ag-GO laminate. As expected, no characteristic peaks of Ag are observed in the XRD pattern of the Ag-GO laminate. The XRD peak at ~10° is attributed to the (001) reflection of the GO laminate, corresponding to an interlayer separation of ~0.9 nm. XRD further affirms that ion adsorption does not alter the lamellar structure of the membrane or impede the diffusion of water between its layers. Figure S2b presents XRD patterns of GO and Ag-GO laminate samples in both air and water. In water, the XRD peaks for both membranes shifted to lower 2θ angles,

suggesting similar_hydration properties for the membranes. The similar interlayer spacing (similar 2θ) for both membranes also confirms that there is no change in the interlayer structure of the laminate due to Ag^+ ion adsorption.

2. Zone of Inhibition/ Kirby Bauer Test

A qualitative evaluation was carried out to assess the bacteriostatic effects of ions released from GO membranes using a zone of inhibition (ZoI)/ Kirby Bauer test. GO membranes incorporated with different ions were placed on a fresh bacterial lawn (S. aureus) streaked on an LB agar nutrient plate. After 24 hours, the ions eluted from the GO membranes were inspected for bacterial inhibition zones. Results showed (**Figure S3**) that the silver ions at both 0.1M and 1M Ag concentrations developed a clear zone devoid of bacterial growth around the samples, whereas no zone of inhibition was observed for copper or zinc ions. The control sample, a bare GO membrane without any ions, also did not exhibit any zone of inhibition, indicating that the GO membrane itself is not bacteriostatic in nature. The ZoI test was performed as a quick screening method to identify which ions could be further tested for long-term antibacterial efficacy.

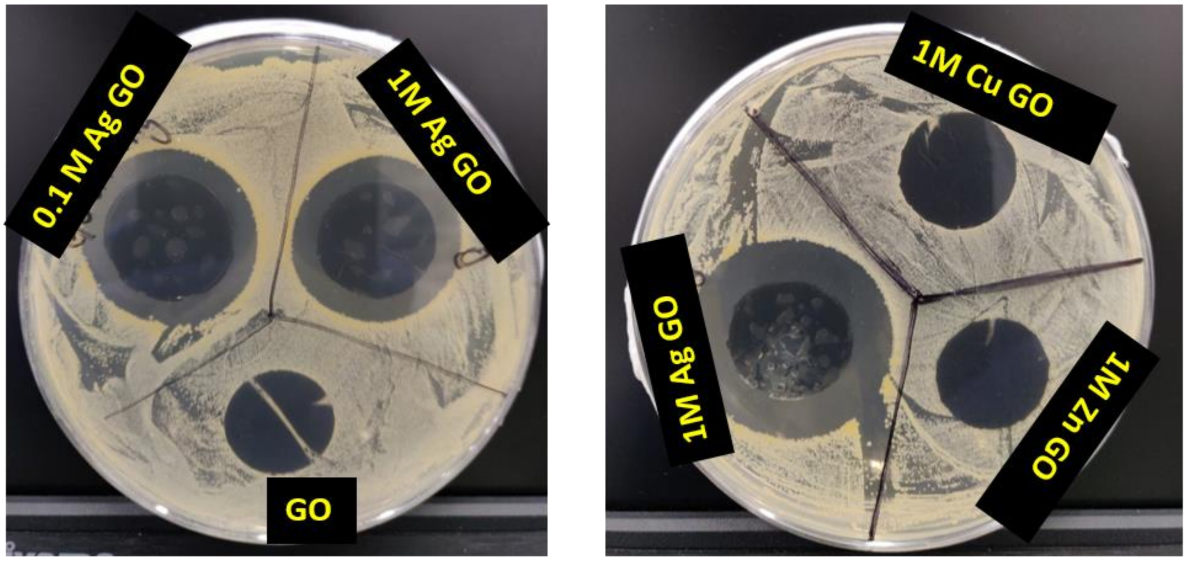

Figure S3: Zone of inhibition test. GO membranes treated with 0.1M and 1M Ag developed a clear, bacteria-free zone on the LB agar plate (left figure). In contrast, GO membranes treated with copper and zinc ions did not have a significant antibacterial effect to prevent bacterial growth and did not form a zone of inhibition, unlike silver (right figure). The control sample of GO showed no zone of inhibition.

3. PE-GO Vs GO free-standing membrane

To mimic a medicated wound dressing application, polyester (PE) membranes were coated with GO by a simple dip-coating mechanism. The GO-PE samples were compared with

freestanding GO membranes for their performance in anti-bacterial activity. The live bacterial counts for these measurements were counted using a flow cytometer (Intellicyt IQ, Sartorius). The multiplying bacterial counts from a starting count of 100 were assessed every 1 hr upto 5hrs into the lag phase of the bacterial growth. **Figure S4** showed that the GO on PE and GO free-standing membranes with and without Ag treatment behaved more or less similarly in nature.

These studies were carried out only in the initial hours of bacterial cell growth primarily because counts at 24hrs may saturate and introduce ambiguity in the results to make any comparison. In the main text, the in-situ OD measurements carried out at 48hrs were performed with GO-PE samples considering ease and practicality.

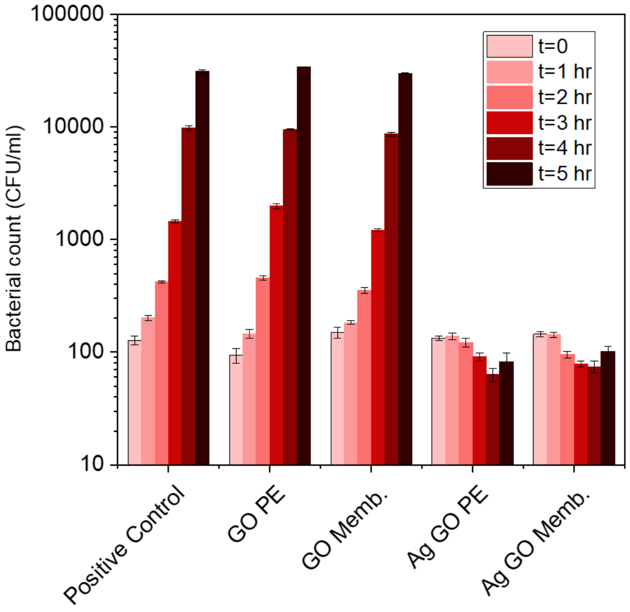

Figure S4: PE-GO Vs GO free-standing membrane. Live bacterial cell count measured in a flow cytometer shows a very good bactericidal for both silver-incorporated GO-PE and free-standing GO membranes. The secondary control samples GO-PE and free-standing GO membranes also had similar performance to the positive control, which was a bare PE membrane. Error bars denote standard deviation from four replicates.

4. <u>Re-culture test for bacterial viability</u>

The viability of bacteria exposed to Ag^{+} ions was studied by reculturing the original bacterial culture that was exposed to the 20-AgGOTi Rod sample until day three. Day three cultures from the positive control, Ti Rod, and 20GOTi Rod were used as control samples. Reculturing experiments were performed by resuspending 100 µL of the day three culture in 3 ml of fresh culture media, and their growth was measured after 24 hours using plate counting methods. **Figure S5** shows the bacterial count after the 24-hour culture, and it demonstrates a difference

of more than 6 orders of magnitude between the control samples and the 20AgGOTi Rod, compared to the four orders of magnitude difference in bacterial concentration in the day three culture (Figure 2d in main text). This suggests that the bacteria exposed to Ag^+ ions are less viable, and their growth is significantly reduced.

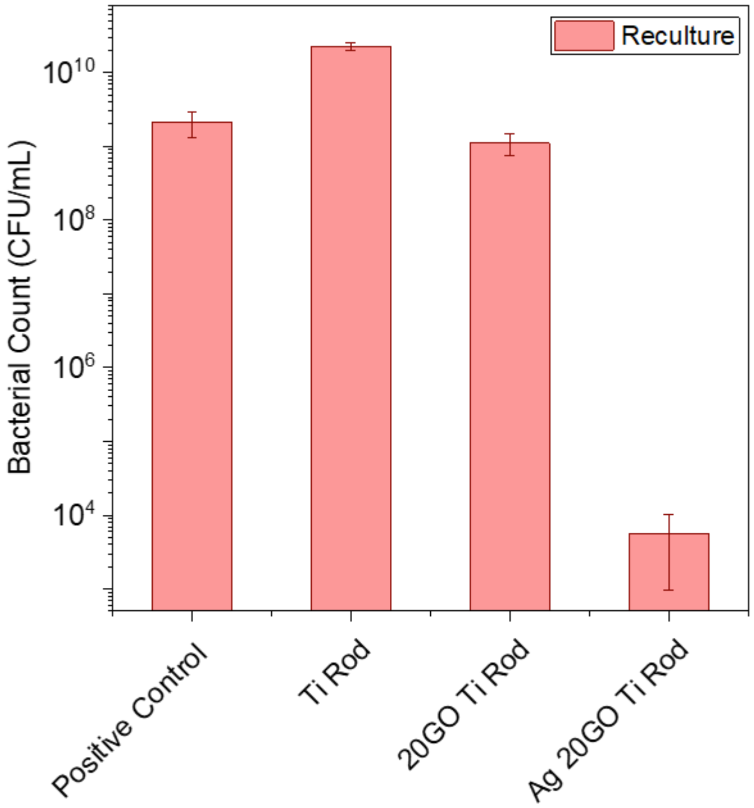

Figure S5: Re-culture test for bacterial viability. Bacterial counts measured from re-culturing samples from Day 3 time point in an all-serum media. 20AgGOTi samples showed near bactericidal counts. Error bars denote the standard deviation from three samples.
